# Effects of Aerobic Exercise Training on MyomiRs Expression in Cachectic and Non-Cachectic Cancer Mice

**DOI:** 10.3390/cancers13225728

**Published:** 2021-11-16

**Authors:** João Lucas Penteado Gomes, Gabriel Cardial Tobias, Tiago Fernandes, André Casanova Silveira, Carlos Eduardo Negrão, Roger Chammas, Patrícia Chakur Brum, Edilamar Menezes Oliveira

**Affiliations:** 1 School of Physical Education and Sport, University of Sao Paulo, Sao Paulo 05508-030, Brazil; joao.gomes@usp.br (J.L.P.G.); gabriel.tobias@usp.br (G.C.T.); tifernandes@usp.br (T.F.); andre.casanova.silveira@usp.br (A.C.S.); carlos.negrao@incor.usp.br (C.E.N.); pcbrum@usp.br (P.C.B.); 2 Heart Institute (InCor), Medical School, University of Sao Paulo, Sao Paulo 05508-030, Brazil; 3 Centro de Investigação Translacional em Oncologia, Instituto do Câncer do Estado de São Paulo, Faculdade de Medicina da Universidade de São Paulo, São Paulo 01246-000, Brazil; rchammas@usp.br

**Keywords:** myomiRs, colon cancer, breast cancer, muscle wasting, cancer cachexia, CT26 cancer model, MMTV-PyMT mice, aerobic exercise training

## Abstract

**Simple Summary:**

Muscle wasting is a symptom of the cancer cachexia closely related to the imbalance between protein synthesis and degradation. MyomiRs are small RNA molecules that do not encode proteins and have the function of regulating protein-coding genes, and in this way, myomiRs can regulate the homeostasis of skeletal muscle cells submitted to physiological or pathological stimulus. Aerobic exercise training (AET) is a nonpharmacological adjuvant treatment to prevent cancer cachexia, improving the patient’s quality of life. MyomiRs are modulated by cancer and AET, as well. Thus, we propose to investigate the effects promoted by AET on circulating and skeletal muscle myomiRs in cachectic and non-cachectic cancer mice. Exercise is a promising therapy for cancer-associated muscle wasting, revealing the importance to understand the molecular mechanisms involved to preserve muscle mass.

**Abstract:**

We investigated the effects of AET on myomiRs expression in the skeletal muscle and serum of colon cachectic (CT26) and breast non-cachectic (MMTV-PyMT) cancer mice models. Colon cancer decreased microRNA-486 expression, increasing PTEN in tibialis anterior muscle (TA), decreasing the PI3K/mTOR protein pathway, body and muscle wasting, fibers’ cross-sectional area and muscle dysfunction, that were not preserved by AET. In contrast, breast cancer decreased those muscle functions, but were preserved by AET. In circulation, the downregulation of microRNA-486 and -206 in colon cancer, and the downregulation of microRNA-486 and upregulation of microRNA-206 expression in breast cancer might be good cancer serum biomarkers. Since the microRNA-206 is skeletal muscle specific, their expression was increased in the TA, serum and tumor in MMTV, suggesting a communication among these three compartments. The AET prevents these effects on microRNA-206, but not on microRNA-486 in MMTV. In conclusion, cancer induced a downregulation of microRNA-486 expression in TA and serum of CT26 and MMTV mice and these effects were not prevented by AET; however, to MMTV, the trained muscle function was preserved, probably sustained by the downregulation of microRNA-206 expression. Serum microRNA-206 is a potential biomarker for colon (decreased) and breast (increased) cancer to monitor the disease evolution and the effects promoted by the AET.

## 1. Introduction

Cachexia is a syndrome associated with several neoplastic diseases. Cancer cachexia is characterized by the progressive loss of lean body mass, with or without the loss of adipose tissue that can be partially but not totally reversed by diet support [1], and the skeletal muscle is the main tissue of protein loss [2]. Cachexia contributes to the reduction in functional capacity and quality of life [2], and these comorbidities account for approximately 20% of cancer deaths [3].

Cancer cachexia, one of the main manifestations of the damage that neoplastic diseases cause, causes muscle atrophy, which is the result of an imbalance between protein synthesis and degradation in skeletal muscle [3]. The reasons for cancer-reduced skeletal muscle mass, cellular mechanism and function are still poorly understood. Additionally, one of the possible mechanisms is through an altered expression of microRNAs in skeletal muscle [4]. MicroRNAs are small non-coding RNAs that negatively regulate their target mRNAs by either promoting messenger decay or by dampening translation [5]. MyomiRs are a set of microRNAs exclusively expressed in skeletal and cardiac muscle, that have been identified as physiological regulators of myogenesis, muscle fiber-type composition, muscle growth and cellular homeostasis [6]. In pathological processes, myomiRs also influence myogenesis and muscle damage repair preventing skeletal muscle injuries and dysfunction [7].

Accumulating evidence shows that the expression of microRNAs in skeletal muscle modifies cancer cachexia in experimental animals and humans [8]. The microRNAs-147-3p, -299a-3p, 1933-3p, 511-3p, 3473d, 233-3p, 431-5p, 665-3p and 205-3p were found to be differently expressed in skeletal muscle in mice with lung cancer [8]. These microRNAs are related to cell survival pathways, inflammatory response, cell cycle, cell development and cell morphology [8]. Narasimhan et al. [9] analyzed small RNAome profiling from skeletal muscle of the cachectic and non-cachectic patients with pancreas or colon cancer. The expression of the microRNAs-let-7d-3p, -3184-3p and -1296-5p were increased in the cachectic patients. These microRNAs were selected for in silico analysis, and target prediction and putative functional annotation revealed targets genes related to adipogenesis, myogenesis, inflammation and immune response [9]. When considered together, these findings highlight that microRNAs are responsible for the development and maintenance of skeletal muscle mass [6,9]. It has been reported that skeletal muscle injury provokes microRNA release from the muscle cells to the plasma. This response suggests circulating microRNAs as biomarkers of physiological or pathological conditions [10]. Furthermore, extracellular microRNAs can have a direct biologic influence in other tissues. For example, microRNA-206, which is a specific skeletal muscle microRNA, was found markedly decreased in estrogen receptors (ER)α positive human breast cancer tissue, and antagomiR-206 treatment inhibits tumor growth in estrogen-dependent breast cancer [11].

Evidence shows that exercise training provokes remarkable adaptations in skeletal muscle in healthy and pathological processes. One of the most marked effects is recovering the muscle protein balance [12,13] that preserves the myofibrillar and sarcoplasmic proteins probably mediated by intracellular mechanisms involving microRNAs’ regulation. Aerobic exercise training (AET) plays an important role in regulating the expression of the microRNAs in skeletal muscle and circulation. AET has therapeutic potential modulating the expression of several altered microRNAs in chronic diseases [14,15,16]. However, whether there is a change in myomiR’s profile in the cancer cachexia is still under-investigated, and the underlying molecular pathways remain to be elucidated.

There is no evidence about the effects of AET on the expression of myomiRs in the cachectic and non-cachectic state associated with cancer. Therefore, our goals are to investigate the profile of myomiRs expressed in CT26 colon cancer cachectic and MMTV-PyMT mammary cancer non-cachectic mice. In addition, to investigate the effects of AET on the myomiRs and skeletal muscle phenotype and function, as well as evaluate whether cancer cachexia and AET are modulating the expression of circulating myomiRs.

## 2. Materials and Methods

### 2.1. Sampling

Balb/c female mice that represent a cachectic model were injected with 1 × 10^6^ of CT26 colon cancer cells (ATCC^®^ CRL-2638™, Manassas, VA, USA). These mice were randomly assigned in colon cancer sedentary (CT26; *n* = 11) and colon cancer trained (CT26 + TR; *n* = 8) groups. A control group was also assigned in wild type sedentary (WT; *n* = 12) and wild type trained (WT+TR; *n* = 5) groups. MMTV (Mouse Mammary Tumor Virus Polyoma Middle T antigen; MMTV-PyMT) c57 female mice that represent non-cachectic transgenic mice with spontaneous tumor appearance were also studied [17]. They were randomly assigned in mammary cancer sedentary (MMTV; *n* = 8) and mammary cancer trained (MMTV+TR; *n* = 8) groups. A control group was assigned in wild type sedentary (WT; *n* = 7) and wild type trained (WT+TR; *n* = 6) groups. The mice were provided from the Medical School of the University of Sao Paulo animal facility. The animals were housed 5 per cage at controlled room temperature (22 °C) with a 12-h dark–light cycle and were fed standard mouse chow and had access to water ad libitum.

### 2.2. Training Protocol

The training protocol was performed on a treadmill according to the protocol developed by Ferreira et al. [18]. Mice were trained 5 days a week for 60-min duration. The exercise intensity was set at 60% of the maximal speed achieved in the maximal exercise test. The CT26+TR groups and the WT+TR were 8 weeks old in the beginning of the training protocol and continued training until they were 14 weeks old. CT26 tumor cells were injected at 12 weeks old. Therefore, the CT26+TR mice remained training cancer-free for 4 weeks and 2 more weeks after tumor cell injection. The MMTV+TR and WT+TR groups were 8 weeks old at the beginning of the training protocol, and the exercise training lasted 8 weeks. Tumors spontaneously appeared in the MMTV and MMTV+TR mice between week 10 and week 13 of age.

### 2.3. Evaluation of the Maximal Physical Capacity

Exercise capacity was assessed by maximal distance running in the maximal exercise test. The exercise test protocol was conducted, as described by Ferreira et al. [18]. Briefly, mice were placed on a treadmill, and speed started at 3 m/min and was increased by 3 m/min every 3 min until exhaustion.

### 2.4. Grip Strength Meter

The grip test was used to evaluate limb traction force and muscular function in the CT26 model. The mice were positioned in the equipment (New Primer, São Paulo, Brazil), suspended by the tail, and stimulated to draw the grid coupled to a dynamometer. Three measurements were performed, and the highest value was selected. The grip test was performed at the end of the training protocol.

### 2.5. Ambulation Test

The ambulation behavioral test was used for the analysis of skeletal muscle function in the MMTV-PyMT model. This test estimates the contractile force and motor coordination. Mice hind legs were placed in contact with non-toxic black paint and then placed inside a rectangular wooden box of 1 m (homemade and lined with white paper) for walking. Subsequently, the stride length was measured.

### 2.6. Tissue Preparation

After euthanasia, the soleus, tibialis anterior (TA), gastrocnemius and plantaris muscles and tibia bone were harvested and weighed. Muscle mass was assessed by the measurement of the ratio of tissue weight in grams to tibia length in millimeters (muscle weight g/mm).

### 2.7. Skeletal Muscle Oxidative Enzyme Activity

To evaluate citrate synthase activity, the gastrocnemius muscle was homogenized in 1 g tissue: 7 µL of extraction buffer containing 50 mM Tris-base, pH 7.4 plus 1.0 mM EDTA at 4 °C. The samples were centrifuged at 3000× *g* for 15 min at 4 °C, and the supernatant was used to perform the enzymatic kinetics. Protein was measured using the method of Bradford et al. [19], using bovine serum albumin as a standard. The maximal activity of the enzyme was determined according to the method of Alp and Newsholme [20] from the quantification of the complex formed between coenzyme A with 5,5′dithiobis 2-nitrobenzoic acid (DTNB) added to the assay buffer, thus forming a yellow complex. The assay buffer was prepared with 100 mM Tris-base, 0.4 mM DTNB, 1.24 mM Acetyl-CoA, 1% (*v*/*v*) Triton X-100 and the homogenate. The reaction was initiated by the addition of 18.9 mM oxaloacetate to the reaction mixture. The enzymatic kinetics was recording and performed at 25 °C for an interval of 10 min, at 412 nm using Victor (Victor3 1420 Multilabel Counter from PerkinElmer, Waltham, MA, USA). The result of the enzyme activity was expressed in nmol·min^−1^·mg protein^−1^.

### 2.8. Analysis of MicroRNA

Frozen TA skeletal muscle samples were used for RNA extraction. Fifteen milligrams of the tissues were homogenized in Trizol (700 μL), and RNA was isolated according to the manufacturer’s instructions (#15596026 Thermo Fisher Scientific, Waltham, MA, USA). Serum RNA was extracted through the miRNeasy Serum/Plasma Kit (#217184 Qiagen, Germantown, MD, USA) following the manufacturer’s instructions. MicroRNAs expression was measured using real-time PCR and the TaqMan MicroRNA Assay (Applied Biosystems, Waltham, CA, USA) as specified by the manufacturer’s instructions. A detailed method was already described by Fernandes et al. [21]. Briefly, real-time PCR reaction included TaqMan Universal PCR master mix II, nuclease-free water, RT product and primers TaqMan MicroRNA Assay for microRNA-486 (Thermo Fisher #2228, Waltham, MA, USA), microRNA-206 (Thermo Fisher #0510), microRNA-133a (Thermo Fisher #2246), microRNA-133b (Thermo Fisher #2247) and microRNA-1 (Thermo Fisher #2064). We also performed U6 snRNA (Thermo Fisher #001973) expression using the same method. The reactions were incubated in a 96-well optical plate. The microRNAs expression was normalized by subtraction of values of U6 expression. Relative quantities of target gene expression in WT vs. other groups were compared after normalization to the values of the reference gene (ΔCT). Fold changes in microRNA expression were calculated using the differences in ΔCT values between the samples (ΔΔCT) and the equation 2^−ΔΔCT^. Results were expressed as % of control groups (WT).

### 2.9. Immunohistochemistry

TA skeletal muscle was harvested and embedded in Tissue-Tek^®^ O.C.T, frozen in isopentane and then in liquid nitrogen. Immunohistochemistry was conducted to analyze the cross-section area; sections (10-micrometer thick) were obtained from TA muscle using a Leica-CM 1850 cryostat (Leica Microsystem, Welzlar, Germany). The muscle sections were fixed with 4% formalin (Sigma-Aldrich, HT501128, São Paulo, Brazil) for 10 min at room temperature, permeabilized in 0.2% Triton X-100 (Biorad, 01-0407, Hercules, CA, USA) and 1% bovine serum albumin (BSA; VWR Amresco Chemicals, E588, Radnor, PA, USA) diluted in phosphate buffer saline (PBS; Sigma-Aldrich, P4417, Saint Louis, MO, USA) for 10 min. Blocking was performed with 10% goat serum (Sigma-Aldrich, G9023, São Paulo, Brazil) in PBS for 45 min. Glass slides were incubated with a solution containing the primary antibody anti-laminin (1:100 dilutions, Thermo fisher, PA1-16730, USA) for delimiting muscle fibers, with 1.5% goat serum in PBS for 1 h 30 min at room temperature. After proper washing, the sections were incubated with a solution with a secondary fluorescent antibody (1:500 dilutions; Alexa Fluor 488 goat anti-rabbit, Life Technologies, A11008, Waltham, MA, USA). The images were captured with a 200× magnification and a 20× objective. The images were recorded on a computer connected to a fluorescent microscope and connected to a photographic system (magnification, 200×) (Leica Qwin, Leica Microsystems, Wetzlar, Germany). The quantification of the cross-sectional area for each fiber was evaluated using the program Image J software (Image J Corporation based in NIH image, Bethesda, MD, USA). The results were expressed in μm^2^.

### 2.10. Immunoblotting

The protein levels PTEN, PI3K, AKT, mTOR, FOXO3a and FOXO3a phosphorylated (pFOXO3a) in the TA muscle were analyzed using Western blotting. Samples were loaded to SDS-PAGE on polyacrylamide gels (6–15%). After electrophoresis, proteins were electro-transferred to a nitrocellulose membrane (BioRad Biosciences, Hercules, CA, USA). Equal loading of samples (30μg) and transfer efficiency were monitored with the use of 0.5% Ponceau S staining of the blot membrane. The blot membranes were incubated with polyclonal antibodies anti-AKT (#9272, Cell Signaling Tech, Danvers, MA, USA), anti-phospho-AKT-SER473 ([pAKT-SER473] #9271s, Cell Signaling Tech, Danvers, MA, USA), anti-PI3K (#ab32569, Abcam, Cambridge, UK), anti-PTEN (#9559, Cell Signaling Tech, Danvers, MA, USA), anti-mTOR (#2972, Cell Signaling Tech, Danvers, MA, USA), FOXO3a (#ab12162, Abcam, Cambridge, UK), FOXO3ap (#ab15478, Abcam, Cambridge, UK) and anti-GAPDH (ab37168, Abcam, Cambridge, UK). The bands were analyzed using Image J software (Image J Corporation based on NIH image, Bethesda, MD, USA). Skeletal muscle GAPDH expression levels were used to normalize the results, which are expressed as a percentage of control expression.

### 2.11. Statistical Analysis

The results are presented as mean ± SEM. Statistical analysis was performed using two-way ANOVA. To indicate how closely two variables, change in relationship to each other, Pearson’s correlation coefficient was used. Probability values of *p* ≤ 0.05 were accepted as statistically significant. The Tukey post hoc test (STATISTICA software; StatSoft, Tulsa, OK, USA) was used for individual comparisons between means when a significant change was observed with ANOVA.

## 3. Results

### 3.1. Effects of Aerobic Exercise Training on Functional Capacity in CT26 Cachectic Model

**Figure 1:**
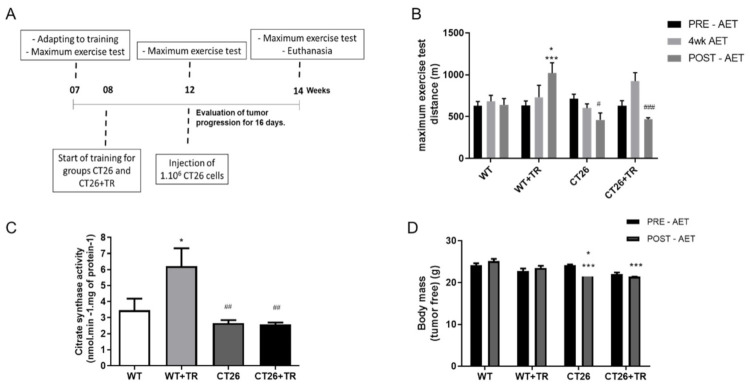
Experimental design, Maximum exercise test, Citrate synthase activity and Body mass to CT26 cachectic mice. (**A**) Experimental design; Groups: WT—wild type sedentary; WT+TR—wild type trained; CT26—colon cancer sedentary; CT26+TR—colon cancer trained. (**B**) Maximal distance running in the maximal exercise test before aerobic exercise training protocol (PRE-AET), four weeks of aerobic exercise training protocol (4wk-AET) and after the end of aerobic exercise training protocol (POST-AET); data are reported as means ± SEM, * *p* < 0.05 vs. WT+TR PRE-AET, WT+TR 4wk AET, WT POST-AET and CT26 4wk AET. *** *p* < 0.001 vs. CT26 POST-AET, CT26+TR POST-AET. ^#^ *p* < 0.05 vs. CT26 PRE-AET and WT POST-AET, ^###^ *p* < 0.01 vs. CT26+TR 4wk AET and WT POST-AET. (**C**) Citrate synthase activity (nmol·min^−1^·mg protein^−1^); data are reported as means ± SEM, * *p* < 0.05 vs. WT and ^##^ *p* < 0.01 vs. WT+TR. (**D**) Tumor free body mass PRE-AET and POST-AET (g); data are reported as mean ± SEM, * *p* < 0.05 vs. CT26 PRE-AET and *** *p* < 0.0001 vs. WT POST-AET.

Aerobic capacity was evaluated using a maximal exercise test at the beginning (PRE-AET), fourth week (4WK-AET) and end of the study (POST-AET) (Figure 1A,B). The results showed no significant change in exercise capacity throughout the study in the WT group PRE-AET (618.9 m ± 47.8) within groups 619.2 m ± 156.6 vs. 618.9 m ± 47.8 vs. 640.1 m ± 75.4, *p* > 0.05 (Figure 1B).

The maximal exercise capacity significantly increased in the WT+TR group at POST-AET (1021 m ± 121.8) compared with PRE-AET (619.2 m ± 156.6, *p* = 0.02), WT POST-AET (640.1 m ± 75.4, *p* = 0.04), CT26 POST-AET (462.5 m ± 82.3, *p* = 0.0004) and CT26+TR POST-AET (470.5 m ± 16.7, *p* = 0.0005).

In the CT26 group, the exercise capacity significantly decreased at POST-AET (462.5 m ± 82.3) compared with the PRE-AET (*p* < 0.05) and WT group POST-AET (640.1 m ± 75.4, *p* = 0.03). The exercise capacity in the CT26+TR group POST-AET (470.5 m ± 16.7) significantly decreased compared with CT26+TR 4wk-AET (971.2 m ± 123.9, *p* = 0.008) and WT POST-AET (640.1 m ± 75.4, *p* = 0.04), but not compared with CT26+TR PRE-AET (645.5 m ± 77.5, *p* > 0.05) (Figure 1B).

Gastrocnemius’ citrate synthase activity was evaluated as a skeletal muscle oxidative metabolism and aerobic training marker. The enzyme activity (nmol·min^−1^·mg protein^−1^) increased by 79% in the WT+TR (6.2 ± 2.5, *p* < 0.05) group compared with WT (3.5 ± 1.7), while it was decreased in the CT26 (2.7 ± 0.4, *p* < 0.003) and CT26+TR (2.6 ± 0.3, *p* < 0.001) groups compared with WT+TR. There were no changes among the WT, CT26 and CT26+TR groups (Figure 1C).

The tumor-free body mass in the CT26 POST-AET (22.3 g ± 0.3, *p* < 0.0001) and CT26+TR POST-AET (21.3 g ± 0.2, *p* < 0.0001) groups were decreased at the end of the training protocol period compared with the WT POST-AET (25.2 ± 0.6) group. Additionally, the tumor-free body mass was decreased in CT26 POST-AET compared with CT26 PRE-AET (24.0 ± 0.9, *p* = 0.03) (Figure 1D).

Altogether, these findings showed that CT26 cachectic mice display exercise intolerance, paralleled by lower body mass and oxidative metabolism, which were not prevented by AET.

### 3.2. Effects of Aerobic Exercise Training on Functional Capacity in MMTV-PyMT Non-Cachectic Model

**Figure 2:**
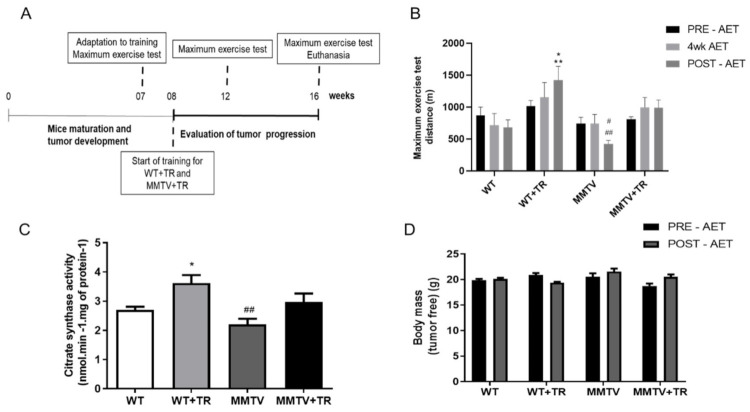
Experimental design, Maximum exercise test, Citrate synthase activity and Body mass to MMTV-PyMT non-cachectic mice. (**A**) Experimental design; Groups: WT—wild type sedentary; WT+TR—wild type trained; MMTV—mammary cancer sedentary; MMTV+TR—mammary cancer trained. (**B**) Maximal distance running in the maximal exercise test before aerobic exercise training protocol (PRE-AET), four weeks of aerobic exercise training protocol (4wk-AET) and after the end of aerobic exercise training protocol (POST-AET); data are reported as means ± SEM, * *p* < 0.05 vs. WT POST-AET and 4wk AET, MMTV PRE-AET and 4wk AET and ** *p* < 0.001 vs. MMTV POST-AET. ^#^ *p*< 0.05 vs. WT POST-AET, vs. MMTV 4wk AET, vs. MMTV+TR POST-AET and 4wk POST-AET. ^##^ *p* < 0.01 vs. MMTV PRE-AET. (**C**) Citrate synthase activity (nmol·min^−1^·mg protein^−1^); data are reported as means ± SEM, * *p* < 0.05 vs. WT and ^##^ *p* < 0.01 vs. WT+TR. (**D**) Tumor free body mass PRE-AET and POST-AET (g); data are reported as means ± SEM.

Aerobic capacity was evaluated using a maximal exercise test at the beginning (PRE-AET), fourth week (4WK-AET) and end of the study (POST-AET) (Figure 2A,B). The results showed no significant change in maximal exercise capacity throughout the study in the WT group PRE-AET (868.6 m ± 134.1), within groups 1016.6 m ± 87 vs. 744.3 m ± 97 vs. 809.9 m± 38, *p* > 0.05 (Figure 2B).

The maximal exercise capacity significantly increased in the WT+TR group at POST-AET (1421.3 m ± 219.7) compared with WT POST-AET (684.7 m ± 115.4, *p* = 0.04) and MMTV POST-AET (423.6 m ± 55.3, *p* = 0.0002), MMTV PRE-AET (*p* = 0.04) and MMTV 4k AET (*p* = 0.03). In the MMTV group, physical capacity significantly decreased at POST-AET compared with PRE-AET (744.3 m ± 97, *p* = 0.004), WT POST-AET (*p* = 0.04) and MMTV+TR POST-AET (990.1 m ± 347, *p* < 0.05). The MMTV+TR group showed no significant change in exercise capacity compared with WT POST-AET (Figure 2B).

Gastrocnemius’ citrate synthase activity in the MMTV and MMTV+TR groups did not differ compared with the WT group. The enzyme activity (nmol·min^−1^·mg protein^−1^) was increased by 33% in the WT+TR (3.6 ± 0.9) group compared with the WT (2.7 ± 0.3, *p* = 0.04) group and 39% compared with the MMTV (2.2 ± 0.6, *p* < 0.001) group. AET restored the enzyme activity in the MMTV+TR (3.0 ± 0.8) group to the same levels of the WT+TR group (Figure 2C). The tumor-free body mass and body was not different among the groups (Figure 2D). Additionally, the body mass was preserved for all the groups.

Therefore, the results show that AET was effective in improving the oxidative capacity and maximal physical capacity, as expected in the WT+TR group. In MMTV-PyMT non-cachectic mice, the physical capacity was impaired in MMTV POST-AET time, also partially impairing the muscle oxidative capacity. However, the AET restored the loss of aerobic capacity and oxidative metabolism.

### 3.3. Effect of aerobic Exercise Training on Skeletal Muscle Mass, Cross-Sectional Area and Function in CT26 Cachectic and MMTV-PyMT Non-Cachectic Mice

**Figure 3:**
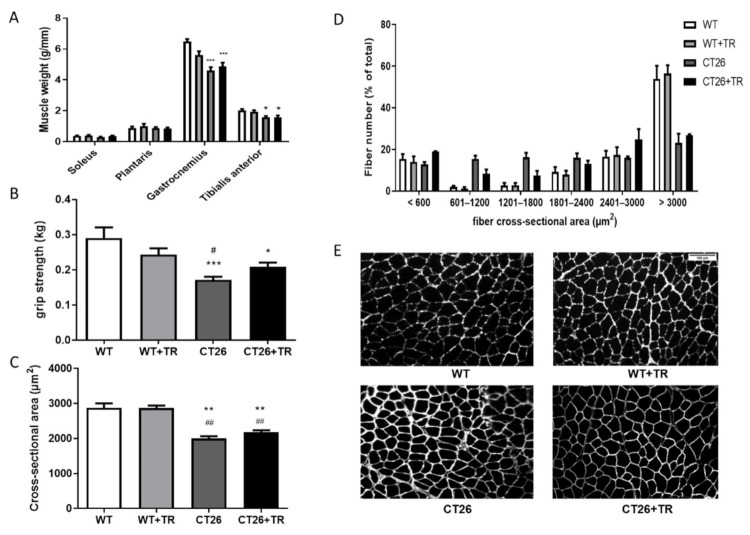
Muscle mass, muscle function and cross-sectional area of CT26 model. (**A**) Soleus, Plantaris, Gastrocnemius and Tibialis anterior muscles mass by tibia length ratio (g/mm); data are reported as means ± SEM, * *p* < 0.05 vs. WT, *** *p* < 0.001 vs. WT. (**B**) grip strength (kg); * *p* < 0.05 vs. WT, *** *p* < 0.001 vs. WT and ^#^ *p* < 0.05 vs. WT+TR. (**C**) Cross-sectional area (µm^2^); ** *p* < 0.01 vs. WT and ^##^ *p* < 0.01 vs. WT+TR. (**D**) percentage distribution of the number of fibers by size. (**E**) Demonstrative Immunohistochemistry image of the cross-sectional area. Groups CT26 cachectic mice: WT—wild type sedentary; WT+TR—wild type trained; CT26—colon cancer sedentary; CT26+TR—colon cancer trained, Scale bar: 100 µm.

Skeletal muscle mass in CT26 cachectic mice was evaluated in soleus, plantar, gastrocnemius and TA. The TA muscle of the CT26 (1.58 g/mm ± 0.1, *p* = 0.01) and CT26+TR (1.57 g/mm ± 0.1, *p* = 0.01) groups showed muscle wasting compared with the WT (2.02 g/mm ± 0.1) group. Similar results were observed in the gastrocnemius muscle where the CT26 (4.59 g/mm ± 0.3, *p* = 0.0001) and CT26+TR (4.83 g/mm ± 0.3, *p* = 0.0001) groups showed muscle wasting compared with the WT (6.42 g/mm ± 0.2) group. There was no effect of AET in restoring the muscle wasting of the TA and gastrocnemius (Figure 3A).

The grip strength was 41% (*p* = 0.0007) decreased in the CT26 group and 28% (*p* = 0.01) in CT26+TR compared with WT. Therefore, there was no effect of AET (Figure 3B).

The cross-sectional area (µm^2^) of the muscle fibers was smaller in the CT26 (2005 ± 243, *p* = 0.01) and CT26+TR (2180.9 ± 296, *p* = 0.01) groups compared with the WT (2879.4 ± 359) and WT+TR (2870.5 ± 345) groups (Figure 3C,E). Figure 3D shows the fibers cross-section area (µm^2^) represented by the total fibers number percentage.

**Figure 4:**
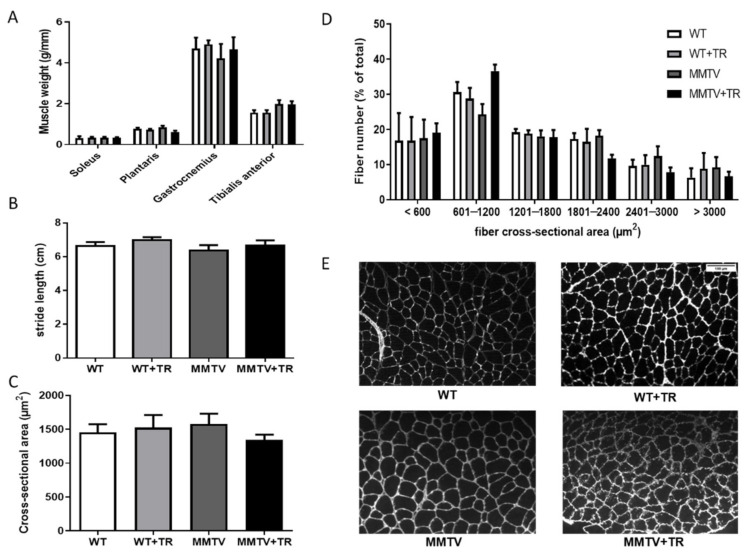
Muscle mass, muscle function and cross-sectional area of MMTV-PyMT model. (**A**) Soleus, Plantaris, Gastrocnemius and Tibialis anterior muscles mass by tibia length ratio (g/mm). (**B**) Stride length (cm). (**C**) Cross-sectional area (µm^2^). (**D**) percentage distribution of the number of fibers by size. (**E**) Demonstrative Immunohistochemistry image of the cross-sectional area. Groups: WT—wild type sedentary; WT+TR—wild type trained; MMTV—mammary cancer sedentary; MMTV+TR—mammary cancer trained, Scale bar: 100 µm.

TA muscles were also evaluated in the MMTV-PyMT non-cachectic mice. There were no significant changes in muscle mass in the MMTV-PyMT non-cachectic mice regardless of exercise training (Figure 4A). The MMTV and MMTV+TR groups and their respective healthy controls showed no change in muscle function test (Figure 4B) or cross-sectional area in muscle fibers (Figure 4C–E). Thus, there were no significant effects of cancer and AET in the MMTV-PyMT model (Figure 4).

### 3.4. MyomiRs Expression Profile of CT26-Cachectic and MMTV-PyMT Non-Cachectic Mice

The expression of microRNAs-1, -133a, -133b, -206 and -486 were evaluated in the TA muscle of both cancer models, CT26 cachectic and MMTV-PyMT non-cachectic mice. In addition, microRNAs-206 and -486 expression was evaluated in the mice serum from both models. No significant differences in myomiRs-1, -133a and -133b expression were found among groups in both cancer models (Figure S1).

**Figure 5:**
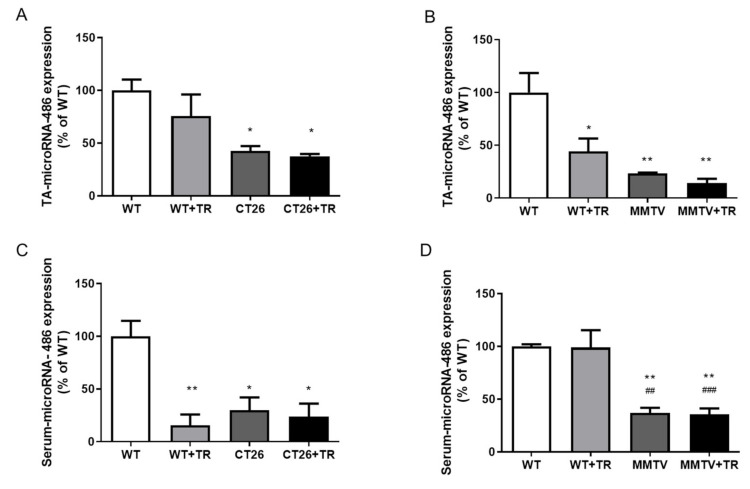
MicroRNA-486 expression in tibialis anterior (TA) skeletal muscle and circulating in CT26-cachectic and MMTV-PyMT-non-cachectic mice. (**A**) CT26 model, skeletal muscle microRNA-486 expression. (**B**) MMTV model, skeletal muscle microRNA-486 expression. (**C**) CT26 model, serum microRNA-486 expression. (**D**) MMTV model, serum microRNA-486 expression. Groups CT26 cachectic mice: WT—wild type sedentary; WT+TR—wild type trained; CT26—colon cancer sedentary; CT26+TR—colon cancer trained. Groups MMTV non-cachectic mice: WT—wild type sedentary; WT+TR—wild type trained; MMTV—mammary cancer sedentary; MMTV+TR—mammary cancer trained. Data are reported as means ± SEM, * *p* < 0.05 vs. WT, ** *p* < 0.01 vs. WT, ^##^ *p* < 0.01 vs. WT+TR and ^###^ *p* < 0.001 vs. WT+TR.

TA microRNA-486 expression was decreased in the CT26 (58%, *p* = 0.03) and CT26+TR (63%, *p* = 0.02) groups compared with the WT (100%) group. No significant difference was found between the WT+TR and WT groups (Figure 5A). The serum microRNA-486 expression was decreased in the WT+TR (85%, *p* = 0.01), CT26 (70%, *p* = 0.006) and CT26+TR (76%, *p* = 0.01) groups compared with WT (100%) (Figure 5C). In the MMTV-PyMT model, TA microRNA-486 expression was decreased in the WT+TR (56%, *p* = 0.03), MMTV (76%, *p* = 0.006) and MMTV+TR (86%, *p* = 0.003) groups compared with WT (100%) (Figure 5B). The serum microRNA-486 expression was decreased in the MMTV (63%, *p* = 0.002) and MMTV+TR (64%, *p* = 0.002) groups compared with WT (100%). No significant difference was found between the WT+TR and WT groups (Figure 5D).

**Figure 6:**
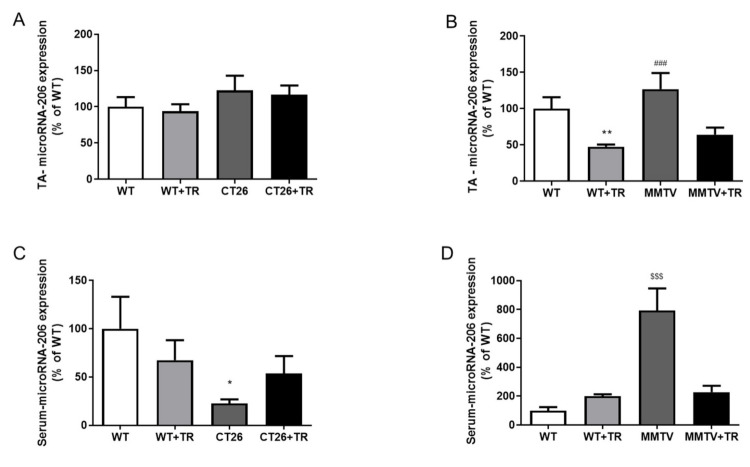
MicroRNA-206 expression in tibialis anterior (TA) skeletal muscle and circulating in CT26-cachectic and MMTV-PyMT-non-cachectic mice. (**A**) CT26 model, skeletal muscle microRNA-206 expression. (**B**) MMTV model, skeletal muscle microRNA-206 expression. (**C**) CT26 model, serum microRNA-206 expression. (**D**) MMTV model, serum microRNA-206 expression. Groups CT26 cachectic mice: W—wild type sedentary; WT+TR—wild type trained; CT26—colon cancer sedentary; CT26+TR—colon cancer trained. Groups MMTV non-cachectic mice: WT—wild type sedentary; WT+TR—wild type trained; MMTV—mammary cancer sedentary; MMTV+TR—mammary cancer trained. Data are reported as means ± SEM, * *p* < 0.05 vs. WT, ** *p* < 0.01 vs. WT, ^###^ *p* < 0.001 vs. WT+TR and MMTV+TR, ^$$$^ *p* < 0.0001 vs. WT, WT+TR and MMTV+TR.

Regarding the TA microRNA-206 expression in the CT26 model, no significant differences were observed among the groups (Figure 6A). The serum microRNA-206 expression was decreased in the CT26 (77%, *p* = 0.04) group compared with the WT (100%) (Figure 6C) group. In the MMTV-PyMT model, TA microRNA-206 expression was increased in MMTV compared with the WT (43%, *p* = 0.02), WT+TR (60%, *p* = 0.002) and MMTV+TR (49%, *p* = 0.001) groups, while it was decreased in the WT+TR (53%, *p* = 0.002) group compared with the WT group (Figure 6B). The serum microRNA-206 expression was increased in the MMTV group compared with the WT (8×, *p* = 0.0003), WT+TR (4×, *p* = 0.0005) and MMTV+TR (3.5×, *p* = 0.0009) groups (Figure 6D).

**Figure 7:**
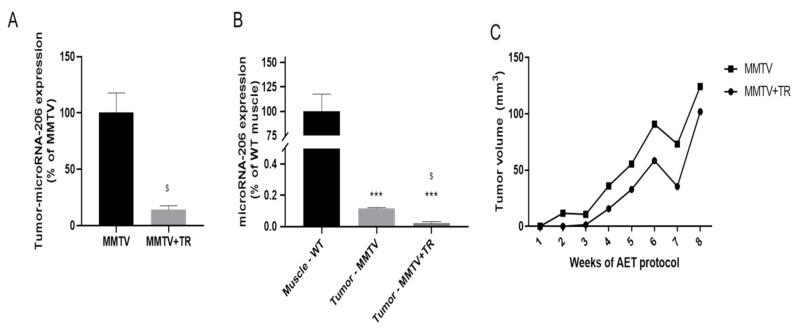
Tumor microRNA-206 expression of MMTV-PyMT model. (**A**) Tumor microRNA-206 expression in MMTV and MMTV+TR groups. (**B**) Comparison of microRNA-206 expression between skeletal muscle and tumors. (**C**) Tumor volume (mm^3^) in MMTV and MMTV+TR groups. Groups MMTV non-cachectic mice: WT—wild type sedentary; WT+TR—wild type trained; MMTV—mammary cancer sedentary and MMTV+TR mammary cancer trained. Data are reported as means ± SEM. *** *p* < 0.05 vs. WT, ^$^ *p* < 0.05 vs. MMTV.

MicroRNA-206, a skeletal muscle-specific myomiR, has attracted attention for its role as a suppressor of breast cancer [11]. Since the microRNA-206 expression was very high in skeletal muscle and mainly in the serum of the MMTV group in the MMTV-PyMT model, we also analyzed the microRNA-206 expression in the tumor. The tumor microRNA-206 expression was strongly decreased in the MMTV+TR (86%, *p* = 0.02) group compared with the MMTV group (Figure 7A). The tumor microRNA-206 expression was very low compared with skeletal muscle (Figure 7B). Although the tumor expression is about a thousand times lower than skeletal muscle, the miR-206 expression measure was able and reliable. However, no significant differences were found in the tumor volume between the MMTV (0.020 ± 0.004 g/mm) and MMTV+TR (0.018 ± 0.008 g/mm) groups (Figure 7C).

**Figure 8:**
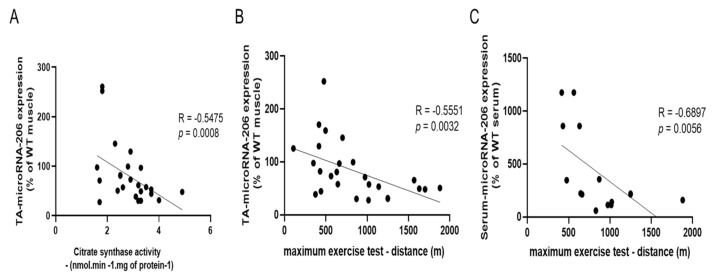
Correlations between microRNA-206 expression in the MMTV-PyMT model with training markers. (**A**) Correlation between TA microRNA-206 expression and citrate synthase activity. (**B**) Correlation between TA microRNA-206 expression and aerobic capacity (distance obtained in the maximum exercise test). (**C**) Correlation between serum microRNA-206 expression and aerobic capacity (distance obtained in the maximum exercise test).

Interestingly, considering the data either from the four MMTV-PyMT model groups, there is a close negative correlation between skeletal muscle microRNA-206 expression and citrate synthase activity (R = −0.5475, *p* = 0.0008; Figure 8A). In addition, there is a close negative correlation between the microRNA-206 expression of the skeletal muscle (R = −0.5551, *p* = 0.0032; Figure 8B) and serum (R = −0.6897, *p* = 0.0056; Figure 8C) and maximal exercise test.

To further analyses the microRNA-206 targets, the PAX3, PAX7 and HDAC4 protein expression were quantified by Western blot in TA muscle. No significant differences were found among the groups of both cancer models (Figure S2).

### 3.5. Effects of AET on MicroRNA-486/PTEN Axis in Skeletal Muscle of CT26-Cachectic and MMTV-PyMT Non-Cachectic Mice

**Figure 9:**
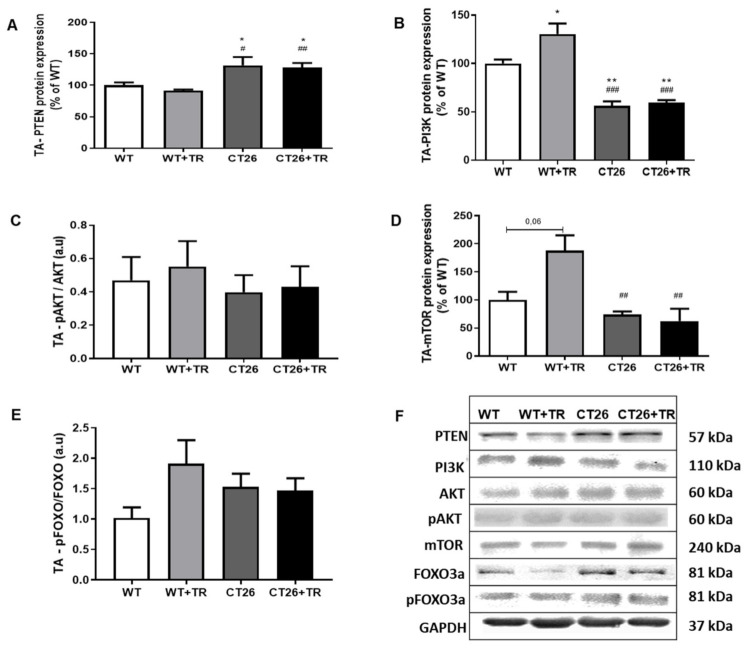
CT26 cachectic mice: PTEN/PI3K/AKT/mTOR pathway proteins expression in tibialis anterior (TA) using Western blot. (**A**) PTEN protein expression. (**B**) PI3K protein expression. (**C**) pAKT and AKT protein expression ratio. (**D**) mTOR protein expression. (**E**) pFOXO3a and FOXO3a protein expression ratio. (**F**) Proteins: PTEN, PI3K, AKT, pAKT, mTOR, FOXO3a, pFOXO3a and GAPDH representative blots. Groups: WT—wild type sedentary (*n* = 8); WT+TR—wild type trained (*n* = 5); CT26—colon cancer sedentary (*n* = 9); CT26+TR—colon cancer trained (*n* = 7). Data are reported as means ± SEM. * *p* < 0.05 vs. WT, ** *p* < 0.01 vs. WT, ^#^ *p* < 0.05 vs. WT+TR, ^##^ *p* < 0.01 vs. WT+TR and ^###^ *p* < 0.001 vs. WT+TR.

PTEN mRNA is a target to microRNA-486 and a key protein that controls the PI3K/AKT/mTOR pathway. To further investigate the CT26 model in this downstream pathway, the TA muscle protein expression was quantified using Western blot. PTEN protein expression was increased in the CT26 (32%, *p* = 0.01) and CT26+TR (28%, *p* = 0.03) groups compared with the WT (100%) group (Figure 9A), and it was also significantly increased compared with the WT+TR group (Figure 9A). PI3K protein expression was increased by AET in the WT+TR (30%, *p* = 0.02; Figure 9B) group and also there was a strong trend to increase mTOR expression (87%, *p* = 0.06; Figure 9D) compared with WT (100%). PI3K protein expression was decreased in the CT26 (44%, *p* = 0.005) and CT26+TR (40%, *p* = 0.02) groups compared with the WT (100%) group and also it was decreased in the CT26 (74%, *p* = 0.0001) and CT26+TR (70%, *p* = 0.0001) groups compared with the WT+TR group (Figure 9B). Likewise, mTOR protein expression was decreased in the CT26 (60%, *p* = 0.008) and CT26+TR (67%, *p* = 0.002) groups compared with the WT+TR group (Figure 9D). In addition, there were no significant expression differences in AKT, phosphorylated AKT (pAKT), FOXO3a and phosphorylated FOXO3a (pFOXO3a) proteins among the groups (Figure 9C and Figure 9E, respectively).

**Figure 10:**
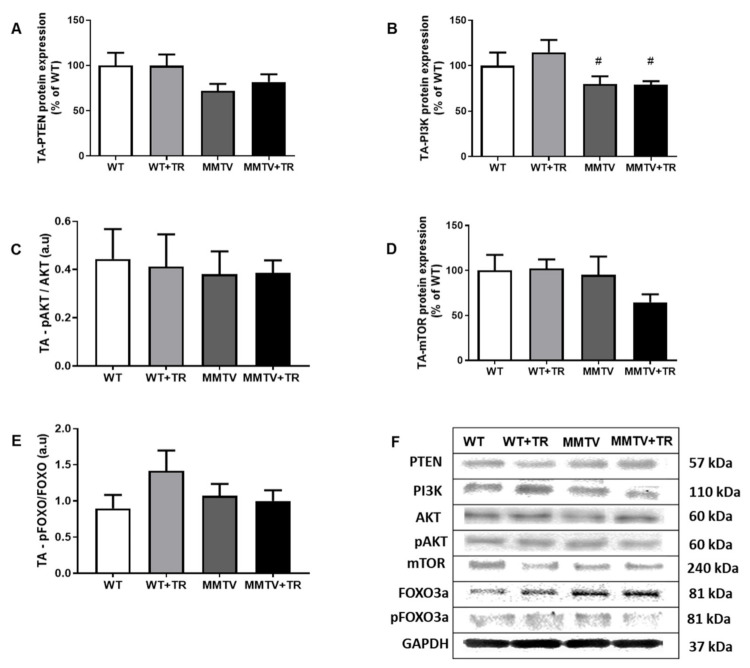
MMTV-PyMT non-cachectic mice: PTEN/PI3K/AKT/mTOR pathway proteins expression in tibialis anterior (TA) using Western blot. (**A**) PTEN protein expression. (**B**) PI3K protein expression. (**C**) ratio between pAKT and AKT protein expression. (**D**) mTOR protein expression. (**E**) pFOXO3a and FOXO3a protein expression ratio. (**F**) Proteins PTEN, PI3K, AKT, mTOR, FOXO3a and pFOXO3a representative blots. Groups: WT—wild type sedentary (*n* = 7); WT+TR—wild type trained (*n* = 5); MMTV—mammary cancer sedentary (*n* = 6); MMTV+TR—mammary cancer trained (*n* = 6). Data are reported as means ± SEM. ^#^ *p* < 0.05 vs. WT+TR.

Despite the fact that MMTV-PyMT mice do not display evidence of muscle cachexia, we further investigated the proteins PTEN/PI3K/AKT/mTOR pathway in TA muscle using Western blot since this signaling pathway may also influence muscle function. PTEN protein expression was not different among the groups (Figure 10A). PI3K protein expression was decreased in the MMTV (30%, *p* = 0.05) and MMTV+TR (31%, *p* = 0.05) groups compared with the WT+TR (100%) group (Figure 10B). In the same way, AKT protein expression was decreased in the MMTV (28%, *p* = 0.05) and MMTV+TR (24%, *p* = 0.05) groups compared with the WT+TR group, although there was no change in pAKT expression among the groups (Figure 10C). In addition, there were no significant differences to mTOR, FOXO3a and pFOXO3a proteins among the groups (Figure 10D,E).

## 4. Discussion

We showed that cancer induced a downregulation of microRNA-486 expression in the TA skeletal muscle and serum of CT26 and MMTV mice. These effects were not prevented by AET. Furthermore, cancer induced a downregulation of microRNA-206 expression in the serum of CT26 mice. Additionally, microRNA-206 was upregulated in the TA skeletal muscle, serum and tumor of the MMTV model, and these effects were prevented by AET. In addition, circulating myomiRs have been suggested as a prognosis cancer biomarker, thus the downregulation of miRNA-486 and -206 in colon cancer, as well as the downregulation miRNA-486 and upregulation of miRNA-206 in breast cancer might be good serum biomarkers to these two kinds of cancer. In addition, the serum miRNA-206 expression of CT26 and MMTV can be a good biomarker of the effects of AET preventing the effects of cancer, as well as of miRNA-206 in TA. According to our knowledge, this is the first study showing the characterization of the myomiRs’ expression in CT26 and MMTV-PyMT animal models submitted to an AET protocol.

The main findings observed in the CT26 model were microRNA-486 expression downregulation in TA skeletal muscle in CT26 and CT26+TR groups, increased protein PTEN levels decreasing the PI3K/Akt/mTOR pathway and AET not being effective in restoring the crucial protein synthesis pathway in skeletal muscle.

We are the first to show the possible role of microRNA-486 in CT26 mice skeletal muscle. Our results suggest that the decreased expression of microRNA-486 leads to a loss in skeletal muscle mass, function and aerobic capacity since it causes impairment in the protein synthesis pathway by targeting the PTEN pathway. These data corroborate a study by Small et al. [22] that inversely showed in vitro and in vivo that PTEN protein levels, the target of microRNA-486, was reduced by microRNA-486 overexpression, which enhances PI3K/AKT signaling [22]. Additionally, decreasing miRNA-486 expression in normal myoblasts results in the impossibility of cell migration and fusion, while overexpression induces muscle regeneration [23]. Consistent with these results, downregulation of microRNA-486 expression was evident in the skeletal muscle of patients with Duchene muscular dystrophy [23].

The decreased expression of microRNA-486 in cancer groups of the MMTV-PyMT model were not followed by changes in PTEN protein expression. However, PI3K and total AKT (not pAKT/AKT) protein expression were downregulated in MMTV and MMTV+TR compared with the WT group. Chen et al. [24] also investigated the effects of microRNA-486 in the MMTV-PyMT model; the authors showed a trend to decrease microRNA-486 expression in skeletal muscle and elevated PTEN expression and lower pAKT, without changes in muscle phenotype [24]. Therefore, our findings also show that the muscle and body mass (Figure 2D and Figure 4A) are preserved in the MMTV group. However, two points should be noticed. Firstly, a low performance at the maximal exercise test can be the first sign of the onset of muscle dysfunction in non-cachectic mice. Secondly, AET prevented this response (Figure 2B). The similarity in microRNA-486 expression in the skeletal muscle of CT26 and MMTV mice cancer groups suggests that downregulation of microRNA-486 may be an early muscle biomarker of impairing skeletal muscle function in MMTV cancer mice.

The microRNA-206 expression was upregulated in the MMTV group and downregulated as an effect of AET in WT+TR and MMTV+TR (Figure 6B). Skeletal muscle has remarkable conditions to adapt and adjust to different stimulus such as AET, activating a variety of signaling pathways and improving metabolic and structural function. In this context, it is well known that AET activates insulin-like growth factor-1 (IGF1), signaling an important anabolic role modulating skeletal muscle mass and metabolism [25]. MicroRNA-206 directly regulates IGF1 gene expression [25,26]. IGF1 is a molecular target validated using a luciferase assay to the microRNA-206 [26,27]. Therefore, microRNA-206 upregulation in the MMTV group (Figure 6B) could be inhibiting the IGF1 signaling pathway corroborating to impair the performance in the maximal exercise test in sedentary breast cancer mice, even the body and skeletal mass, fiber number, cross-sectional area and function were still preserved (Figure 2D and Figure 4). Corroborating these results, we observed a negative correlation between microRNA-206 expression and muscle oxidative metabolism and physical capacity (Figure 8A–C). These findings suggest an association between microRNA-206 expression and physical performance and lead speculations regarding microRNA-206 expression being a rate-limiting step in this process or at least having some effect on skeletal muscle metabolism and physical performance in maximal exercise test (Figure 2B). Importantly, changes of microRNA-206 expression have been associated with skeletal muscle disorders such as Duchenne muscular dystrophy [28].

Therefore, our results suggest that the downregulation of microRNA-486 and the upregulation of microRNA-206 are contributing to the impairment in exercise capacity and muscle functioning in the MMTV group that is prevented by AET with the downregulating of microRNA-206 expression in the MMTV+TR group. This result is probably due to the fact that microRNA-206 is a skeletal-muscle-specific microRNA inducing more robust effects than a non-muscle specific microRNA.

MyomiRs, in turn, are a class of microRNAs with enriched expression in skeletal and cardiac muscles that act by controlling myogenesis, homeostasis, muscle metabolism and regeneration [6]. In terms of function, myomiRs target genes related with skeletal muscle differentiation and growth [6,29,30]. To this end, we also analyzed the microRNA-1, -133a and -133b; however, their expressions were not modified to any groups (Figure S1). Furthermore, to investigate whether the myomiRs would promote a possible skeletal muscle differentiation, we analyzed the expression of the predict genes to fiber myogenesis, histone deacetylase 4 (HDAC4) and paired box transcript factors 7 (Pax 7) and 3 (Pax 3), the expressions of were not modified as well (Figure S2). Thus, other myogenic pathways [31] that were not investigated here could be involved in preserving the skeletal muscle mass in non-cachectic MMTV-PyMT breast cancer mice.

CT26 and MMTV sedentary mice showed low aerobic capacity, which was preserved by the AET (Figure 1B and Figure 2B). These results are not related to the changes in energy consumption for both models (data not shown). Additionally, the low aerobic capacity is a strong predictor of mortality in cancer patients [32]. Neil-Sztramko et al. [33] showed that in patients with breast cancer, the aerobic capacity and strength of the upper limbs are severely decreased before, during and after chemotherapy treatment, which is independent of age. The aerobic capacity impairment may be associated with the development of other comorbidities [33]. Thus, AET could be an important coadjutant non-pharmacological treatment mainly for breast cancer patients. In the CT26 model, the low aerobic capacity could be associated with the muscle wasting and of the fiber cross-sectional areao, oxidative metabolism and muscle function that was not preserved by AET, although the evidence in the literature describing the mechanisms by which the AET is effective to mitigate skeletal muscle wasting, as reviewed by Alves et al. [34], preserves the individual’s quality of life with chronic diseases.

Circulating microRNAs promote cell to cell and tissue to tissue communication in an autocrine, paracrine and endocrine manner. The serum microRNA-486 expression was downregulated in the CT26, CT26+TR, MMTV and MMTV+TR groups (Figure 5C,D) compared with their respective healthy WT groups, and a similar expression was observed in the TA skeletal muscle (Figure 5A,B). These results suggest a link between the effects promoted by the tumor on skeletal muscle and the circulating of microRNA-486 levels. These data reinforce microRNAs as circulating non-invasive biomarkers for cancer [35,36], and the microRNA-486 can be used to monitor the muscle dysfunction and cachexia evolution in colon cancer.

In the CT26 cancer colon mice, the serum microRNA-206 expression in the CT26 group was downregulated compared to all the other groups (Figure 6C). Liu et al. [37] confirmed microRNA-206 as an independent prognostic indicator for colorectal cancer. Patients with low microRNA-206 expression had a poor prognosis and worse survival [37]. The authors demonstrated that serum microRNA-206 was a good diagnostic marker for discriminating colorectal patients from healthy controls. MicroRNA-206 levels were increased in the blood samples of colorectal patients who received surgical treatment [37]. Similarly, our study showed that AET prevented the low circulating microRNA-206 expression in WT+TR and CT26+TR compared to the CT26 group (Figure 6C), suggesting the AET as a potential coadjutant non-pharmacological therapy to colon cancer. Additionally, circulating microRNA-206 expression has highlighted its potential as a diagnosis and prognostic biomarker to monitor the effects of AET for colorectal cancer patients.

In the MMTV group, the TA and serum microRNA-206 expression was significantly increased compared with the other groups (Figure 6B,D). These results suggest that the microRNA-206 downregulation in TA skeletal muscle promoted by AET could be important to preserve maximal exercise capacity (Figure 2B) and metabolism (Figure 2C), since a negative correlation between TA microRNA-206 expression and citrate synthase activity was observed (Figure 8A). An interesting result was observed in the MMTV group; the circulating microRNA-206 expression was eight times higher than skeletal muscle (Figure 6B,D) and the serum and TA microRNA-206 were both negatively correlated with the maximal exercise test (Figure 8B,C). Therefore, the increase in TA microRNA-206 expression was correlated with the decreased muscle oxidative capacity impairing the maximal aerobic capacity, which is also corroborated by the increased circulating microRNA-206 expression.

For a long time, microRNA-206 has been reported as having a tumor suppressor role in breast cancer [11]. Once the serum microRNA-206 expression was very high in the MMTV group and was decreased by AET, based on the hypothesis that microRNA-206 could be a tumor suppressor [11], we also evaluate the microRNA-206 expression in the tumor samples (Figure 7). The tumor microRNA-206 expression was downregulated by AET in MMTV+TR compared to the MMTV group (Figure 7A,B). However, the tumor volume was not modified as an effect of the AET (Figure 7C). Since the microRNA-206 expression is exclusive of skeletal muscle, these results together suggest a link among the skeletal muscle, circulation and tumor that can be modulated by the AET. Thus, breast cancer mice increased the microRNA-206 expression in these three tissues, and AET prevented this effect. These data reinforce microRNAs as non-invasive circulating biomarkers, and microRNA-206 expression is suggested as an independent prognostic indicator for breast cancer and as a tool to monitor the beneficial effect of AET.

It is noteworthy that the results related to microRNA-206 expression in breast cancer and the effects of AET are unclearly established, sometimes they are contradictory, mainly regarding the effects of AET. MicroRNA-206 expression is linked to many malignancies and plays an important role in several cancers’ tissues [38,39]. In breast cancer cell lines and breast cancer tissues, microRNA-206 expression was upregulated when compared to adjacent normal tissues. The upregulation of microRNA-206 expression promotes breast cancer cell invasion, migration, proliferation and colony formation in vitro, and the in vivo inhibition of tumor formation by anti-microRNA-206 in mice confirmed the suppression of proliferation [40]. Our study shows that AET significantly decreases the tumor microR-206 expression compared with sedentary mice (Figure 7A,B); however, the tumor volume was not modified (Figure 7C). Conversely, it was found that a lowered microRNA-206 expression in the patient’s breast cancer tissues was related to larger tumor size and more advanced clinical stage [41]. Recently, Calzia et al. [42] showed myoblasts stimulated by gravity compared with a lack of gravity, mimicking a lack of physical activity, and released microvesicles containing microRNA-206, inhibiting breast cancer cell growth [42]. Therefore, exercise training regulates several physiological processes; however, little is known regarding the mechanisms by which microRNAs regulate the tumor microenvironment, via endocrine and paracrine systems, modulated by exercise training from the distant tissues, for example, skeletal muscle. Nevertheless, this finding encourages further investigation in future studies.

## 5. Conclusions

In conclusion, our results suggest mechanisms by which myomiRs can regulate skeletal muscle mass and function in colon and breast cancer. They also provide evidence for the effects of the AET on circulating myomiRs. CT26 (colon cancer, cachectic) mice, microRNA-486 downregulation results in TA and gastrocnemius cachexia and muscle dysfunction. These effects were not prevented by AET. Our findings showed, for the first time, the microRNA-486 expression in skeletal muscle cachexia resulting from colon cancer in mice. These results suggest that treatment with mimic-microRNA-486 may contribute to preserving the skeletal muscle mass and individual quality of life. The MMTV-PyMT (breast cancer, non-cachectic) mice, downregulation of microRNA-486 expression and upregulation microRNA-206 in the MMTV cancer group corroborate the impairment in the skeletal muscle performance, although the muscle mass is still preserved. In contrast, AET decreases microRNA-206 expression in the MMTV model and improves aerobic capacity. These results suggest that microRNA-486 downregulation in the MMTV group can be the first signaling and early biomarker of muscle dysfunction, since it is also downregulated in the cachetic model, also suggesting that the effects of the microRNA-muscle-specific are more prevalent than a non-muscle-specific microRNA.

Circulating microRNA-486 expression levels were downregulated in the sedentary and trained colon and breast cancer mice. Similar results were observed in the TA skeletal muscle, suggesting a link between the effects promoted by the tumor on skeletal muscle and circulating of microRNA-486 levels. The circulating microRNA-206 expression levels were very low in CT26 cancer mice, suggesting microRNA-206 as an independent prognostic indicator for colorectal cancer. AET increased the expression in both trained groups (Figure 6C). On the other hand, the circulating, skeletal muscle and tumor microRNA-206 expression was very high in the MMTV mice and normalized by AET. Our results on circulating microRNAs reveal microRNA-206 as a potential biomarker for colon (decreased) and breast (increased) cancer to monitor the disease evolution and the effects promoted by the AET.

CT26 and MMTV mice have low aerobic capacity, which is a strong predictor of poor prognosis. More importantly, this change can be preserved by AET. Thus, AET might be an important non-pharmacological strategy for the treatment of patients with breast and colon cancer. MicroRNAs have the potential to be cancer biomarkers that can be modulated by AET. This is a promising area for future studies.

## Supplementary Materials

The following are available online at https://www.mdpi.com/article/10.3390/cancers13225728/s1, Figure S1: skeletal muscle microRNAs expression in CT26 cachectic and MMTV-PyMT non-cachectic mice, Figure S2: skeletal muscle proteins expression in CT26 cachectic and MMTV-PyMT non-cachectic mice.

## Abbreviations

The following abbreviations are used in this manuscript: AET—Aerobic exercise training; WT—Group wild type sedentary; WT+TR—Group wild type trained; CT26—Group cancer CT26 sedentary; CT26+TR—Group cancer CT26 trained; MMTV—Group cancer MMTV-PyMT sedentary; MMTV+TR—Group cancer MMTV-PyMT trained; qPCR—Quantitative real-time polymerase chain reaction; TA—Tibialis anterior; PTEN—Phosphatase and tensin homologue; PI3K—Phosphoinositide 3-kinase; mTOR—Mechanistic target of rapamycin; AKT—Protein kinase B; pAKT—Protein kinase B phosphorylated; ER—Estrogen receptors; DTNB—5,5-dithio-bis-(2-nitrobenzoic acid); FOXO3a—Forkhead box O3; pFOXO3a—Forkhead box O3 phosphorylated; SEM—Standard error of the mean; PRE-AET—Time before aerobic-exercise-training protocol; 4WK-AET—Time after four weeks of aerobic-exercise-training protocol; POST-AET—Time after aerobic-exercise-training protocol; HDAC4—histone deacetylase 4; PAX7—Paired box transcript factors 7; PAX3—Paired box transcript factors 3; RNA—Ribonucleic acid; mRNA—Messenger ribonucleic acid.
