# Supplementary material for: Effects of Aerobic Exercise Training on MyomiRs Expression in Cachectic and Non-Cachectic Cancer Mice
Source: Cancers (Basel). 2021 Nov 16;13(22):5728. doi: 10.3390/cancers13225728 (PMC8616427; doi:10.3390/cancers13225728)
Supplement: Supplementary file 1 [file cancers-13-05728-s001.zip › cancers-1353303-supplementary.pdf]

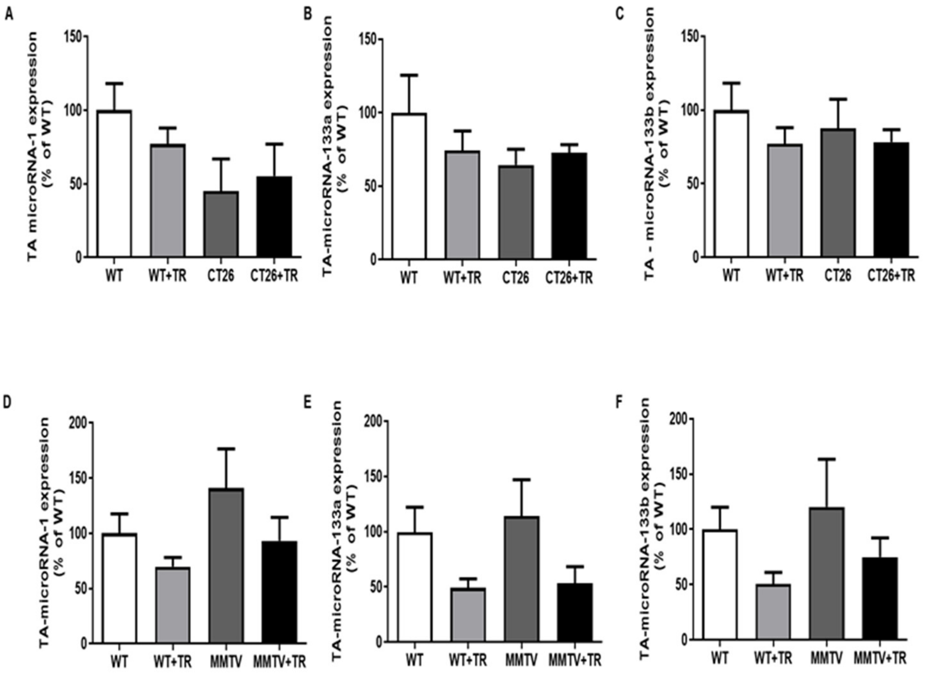

Figure S1. skeletal muscle microRNAs expression in CT26 cachectic and MMTV-PyMT non-cachectic mice. (**A**) microRNA-1 expression in CT26 model; (**B**) microRNA-133a expression in CT26 model; (**C**) microRNA-133b expression in CT26 model (**D**) microRNA-1 expression in MMTV model; (**E**) microRNA-133a expression in MMTV model (**F**) microRNA-133b expression in MMTV model. Groups CT26 cachectic mice: W-wild type sedentary; WT+TR- wild type trained; CT26 - colon cancer sedentary; CT26+TR-colon cancer trained. Groups MMTV non-cachectic mice: WT-wild type sedentary; WT+TR-wild type trained; MMTV-mammary cancer sedentary; MMTV+TR-mammary cancer trained. Data are reported as means ± SEM.

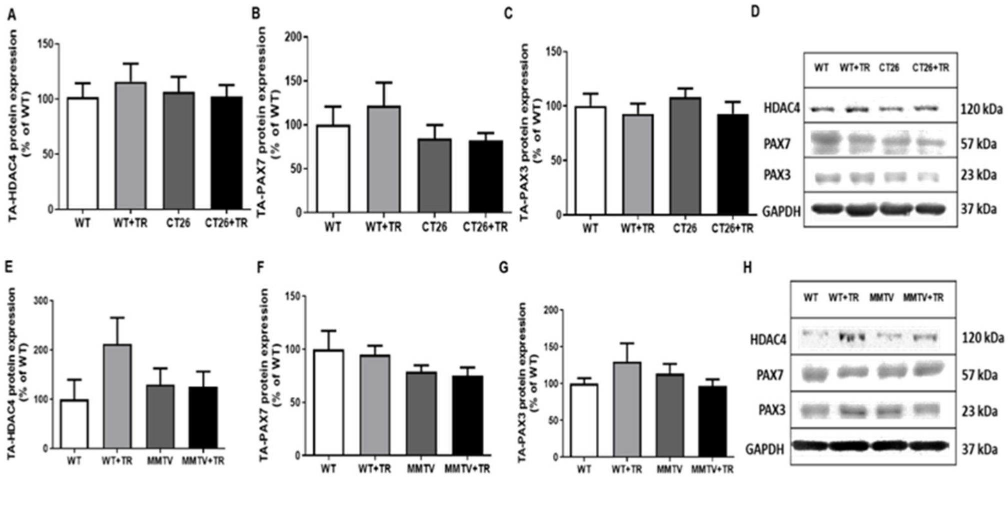

Figure S2. skeletal muscle proteins expression in CT26 cachectic and MMTV-PyMT non-cachectic mice. (**A**) HDAC4 protein expression in CT26 model; (**B**) PAX7 protein expression in CT26 model; (**C**) PAX3 protein expression in CT26 model (**D**) representative blots of PAX7, HDAC4 and PAX3 expression in CT26 model; (**E**) PAX7 protein expression in MMTV model (**F**) HDAC4 protein expression in MMTV model (**G**) PAX3 protein expression in MMTV model (**H**) representative blots of PAX7, HDAC4 and PAX3 expression in MMTV model. Groups CT26 cachectic mice: W-wild type sedentary; WT+TR- wild type trained; CT26 - colon cancer sedentary; CT26+TR-colon cancer trained. Groups MMTV non-cachectic mice: WT-wild type sedentary; WT+TR-wild type trained; MMTV-mammary cancer sedentary; MMTV+TR-mammary cancer trained. Data are reported as means ± SEM.
